# Cryo-EM structures of bacteriophage T4 portal-neck connector complexes reveal a viral genome retention mechanism involving a host component

**DOI:** 10.21203/rs.3.rs-6853951/v1

**Published:** 2025-06-27

**Authors:** Lin Han, Qiyu Mao, Jingen Zhu, Xiaohui Jin, Xiaodan Wang, Zhimin Liu, Xiaorong Wu, Qianglin Fang, Andrei Fokine, Venigalla B Rao, Zhenguo Chen, Lei Sun

**Affiliations:** 1 Shanghai Fifth People’s Hospital, Shanghai Institute of Infectious Disease and Biosecurity, and Institutes of Biomedical Sciences, Fudan University, Shanghai 200032, China.; 2 Bacteriophage T4 Medical Research Center, Department of Biology, The Catholic University of America, 620 Michigan Ave. N.E., Washington, DC 20064, USA.; 3 School of Public Health (Shenzhen), Sun Yat-sen University, Shenzhen, Guangdong, 518107, China.; 4 Department of Biological Sciences, Purdue University, 240S. Martin Jischke Drive, West Lafayette, Indiana 47907-2032, USA.; 5 Lead contact

**Keywords:** Bacteriophage T4, neck assembly, *E. coli* Hfq, genome-gate, sealing packaged head, genome retention

## Abstract

Bacteriophage T4 is a model for tailed viruses, the most abundant biological entities on Earth. During virion assembly, the DNA genome is tightly packed inside the head, which then attaches to tail via a portal-neck connector. Keeping this pressurized head leak-proof during these transactions is a challenge because even a few basepairs leakage could disrupt assembly, but the mechanisms are not understood. Here, we solved the structures of *in vitro*-assembled portal-neck complexes and found a double “genome-gate” in the neck that plugs the packaged head. The first gate is formed by gp14 hexamer, while the second, unexpectedly, consists of a host component, Hfq hexamer. Furthermore, Hfq stabilizes the neck and facilitate its correct docking onto the head. Thus, a pre-assembled, gp13/gp14/Hfq neck complex awaits genome packaging completion, triggers portal conformational changes and packaging motor ejection, which exposes binding sites for the neck to securely seal the pressurized head.

## Introduction

The bacteriophage (phage) T4 is a contractile tailed phage that infects *Escherichia coli* bacterium. It is a member of *myoviridae* family, which constitutes one the most abundant and widely distributed viruses on Earth^1^. Historically, phage T4 has served as an important model to elucidate the fundamental principles of molecular biology, and as a prototypical virus to tease out the mechanisms of icosahedral virus assembly and infection. ^2,3^ Indeed, many mechanistic parallels were discovered between the prokaryotic T4 phage and the eukaryotic herpes virus assembly and genome packaging^4,5^. T4 has also emerged as a versatile platform to design next generation vaccines and gene therapies^6–9^.

T4 first assembles a dodecameric, mushroom-shaped, portal ring structure that acts as the initiator of head (capsid) assembly through interactions with the major capsid protein gp23 and the major scaffold protein gp22. A prolate icosahedral shell made of gp23 assembles around the scaffolding core made of gp22, with the dodecameric portal forming the unique vertex. The rest of the eleven vertices are occupied by the pentameric gp24. Later, the portal serves as a docking site for assembling a pentameric DNA packaging motor which powers translocation of the DNA genome into the capsid through a central channel^10–13^. After encapsidating ~171-kb (“headful”) genome, packaging is terminated and the motor is ejected, allowing the portal to interact with the neck proteins gp13 and gp14. Sequential assembly of neck, tail, and tail fibers then generate an infectious virion. During infection, the virion delivers its genome into the host cell through a tunnel formed by portal, neck, and tail^13–15^.

The structural components of T4 have been extensively investigated. Numerous high-resolution structures have been determined by X-ray crystallography and/or cryo-electron microscopy (EM), including that of the head, the portal, the baseplate, and the tail^2,15–19^. However, the structure of the neck is yet to be resolved. Neck is a critical component because, in addition to connecting the packaged head to the tail, it acts as a plug (seal) to prevent leakage of the packaged DNA^20–24^. The timing and integrity of this seal are crucial because the highly acidic genome compacted to near crystalline density (~500 mg/ml) creates tremendous pressure, ~25–35 atm^25,26^ or 5–7 times the pressure in a champagne bottle. Packaging termination, motor ejection, and neck and tail attachment must be seamlessly coordinated while keeping the head leak-proof. Otherwise, the internal pressure could expel the DNA resulting in abortive assembly. Thus, neck assembly is one of the most vulnerable steps in virion assembly^10,27,28^. Though several neck structures have been determined,^29–33^ they are all in the mature phage, the finished product. The conformational dynamics of how a plug is created and unsealed, and the mechanics of how the genome is fully retained inside the head remain poorly understood in any phage or virus.

Here, we developed an *in vitro* system and successfully assembled a variety of neck (gp13/gp14) and portal-neck (gp20/gp13/gp14) complexes. To our surprise, we discovered that a previously unknown protein, identified as the *E. coli* Hfq (Host Factor for phage Qβ RNA replication), participates in T4 neck assembly and in creating a tight seal. Hfq, originally discovered as a *h*ost *f*actor involved in phage *Q*β replication, is an abundantly expressed hexameric protein that acts as a pleotropic regulator of gene expression in bacteria. We solved a series of high resolution cryo-EM structures, including gp13 (dodecamer)/gp14 (hexamer) complexes with and without Hfq (hexamer), to 2.66Å and 3.47 Å resolution, respectively, and the gp20 portal dodecamer/gp13 dodecamer/gp14 hexamer/Hfq hexamer complex to 2.96 Å resolution. These structures reveal a phage T4 neck containing *two* closed genome-gates (“double genome-gate”); one formed by “stopper loops” of gp14 projected into the lumen of neck channel, and a novel second gate formed by the binding of Hfq hexamer to gp14 in the neck channel. This distinctive and reinforced double gate structure, which creates a strong seal of the packaged head, and the conformational transitions informed by structural analyses, suggest a detailed genome retention mechanism that securely contains the packaged genome in the pressurized head and ensures efficient assembly of an infectious virion.

## Results

### *In vitro* assembly of T4 neck-portal connector complexes

**Fig. 1 |:**
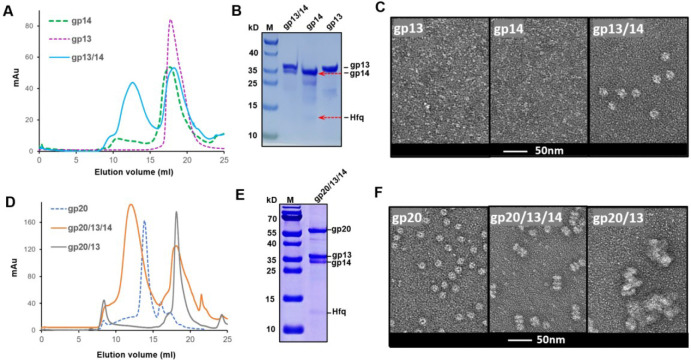
*In vitro* assembly of bacteriophage T4 neck-portal complexes. (A) Size-exclusion chromatography (SEC) shows the elution profiles of gp13, gp14, and the gp13/gp14 complex. (B) SDS-PAGE analysis of the peak fraction of SEC. Hfq was observed in the purified gp14 samples. (C) Negative-stain electron microscopy images of gp13, gp14, and the gp13/gp14, showing that individual gp13 and gp14 exist as monomers, while the gp13/gp14 complex assembles into a larger structure. The scale bar represents 50 nm. (D) SEC profiles of gp20, gp20/13/14, and the gp20/gp13 complex. (E) SDS-PAGE analysis of the SEC peak fraction of gp20/13/14 complex. (F) Negative-stain electron microscopy images of gp20, gp20/13/14, and the gp20/gp13 complex, showing that gp20/13/14 form larger complex, while gp13 alone induces aggregation of gp20. The scale bar represents 50 nm.

To investigate the structure and mechanism of the T4 neck-portal connector complex, we overexpressed gp20, gp13, and gp14 individually in *E. coli*, purified the proteins (Fig. 1A and 1D), and visualized the samples by transmission electron microscopy (TEM). Negative-staining EM showed that while gp20 formed dodecamers as expected (Fig. 1F), gp13 and gp14 existed as monomers (Fig. 1C). In contrast, when we mixed the *E. coli* lysates to allow the assembly to occur *in vitro* and then purified the proteins, we observed co-purification of a variety of new oligomeric complexes.

Mixing gp13 and gp14 lysates resulted in the assembly of ring structures (Fig. 1A–C), whereas mixing of all three lysates of gp20, gp13 and gp14 resulted in even larger ring structures containing all three proteins (Fig. 1D–F). In contrast, mixing gp13 with gp20 did not produce ring structures but instead resulted in aggregates (Fig. 1F). These results demonstrate *in vitro* assembly of post-packaging neck connector structures, and that the neck protein gp13 preassembles first with gp14 forming stable oligomeric rings before attaching to the dodecameric portal.

### Involvement of a host factor, Hfq, in T4 neck assembly

Surprisingly, we observed an additional ~12 kDa protein band in the purified preparations containing gp14 (Fig. 1A–B). LC–MS/MS analysis identified this gp14-associated band as the Hfq protein of *E. coli*. Hfq is an abundant *E. coli* protein and originally discovered as a host factor for phage Qβ replication. It was later characterized as a pleiotropic regulator in *E. coli* and is commonly found in most bacterial genomes^34,35^. Although Hfq primarily binds to RNA, it can also interact with a wide range of nucleic acids including DNA, and regulates transcription, genome stability, and mRNA decay^36–38^.

**Extended Data Fig. 1 |:**
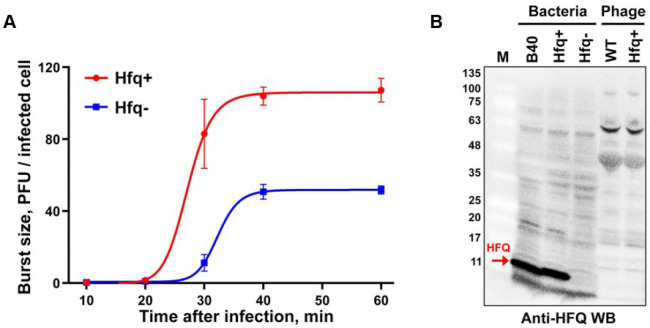
*In vivo* infection assay, assessing the role of Hfq in T4 phage assembly. (A) Phage yield expressed as pfu produced per infected cell in Hfq+ and Hfq− *E. coli* infections. (B) Detection of Hfq by Western blotting using Hfq-specific polyclonal antibodies. Hfq is not present in WT T4 phage, or in *hfq*+ phage. The *hfq+* was constructed by inserting the *hfq* gene into T4 genome under the control of a strong late promoter of *soc* gene.

To further assess the role of Hfq in T4 phage assembly, we performed *in vivo* infection assays. The results showed that phage yield of wild-type (WT) T4 phage on *hfq*-minus *E. coli* NM 22565^39^ is ~2-fold lower than that from isogenic *hfq*-plus *E. coli* NM22540 (Extended Data Fig. 1A), implicating the participation of Hfq in T4 phage assembly. However, its role appears to be transient because Hfq is absent in the mature T4 phage virion (Extended Data Fig. 1B).

### Cryo-EM structures of T4 neck complexes

**Extended Data Fig. 2 |:**
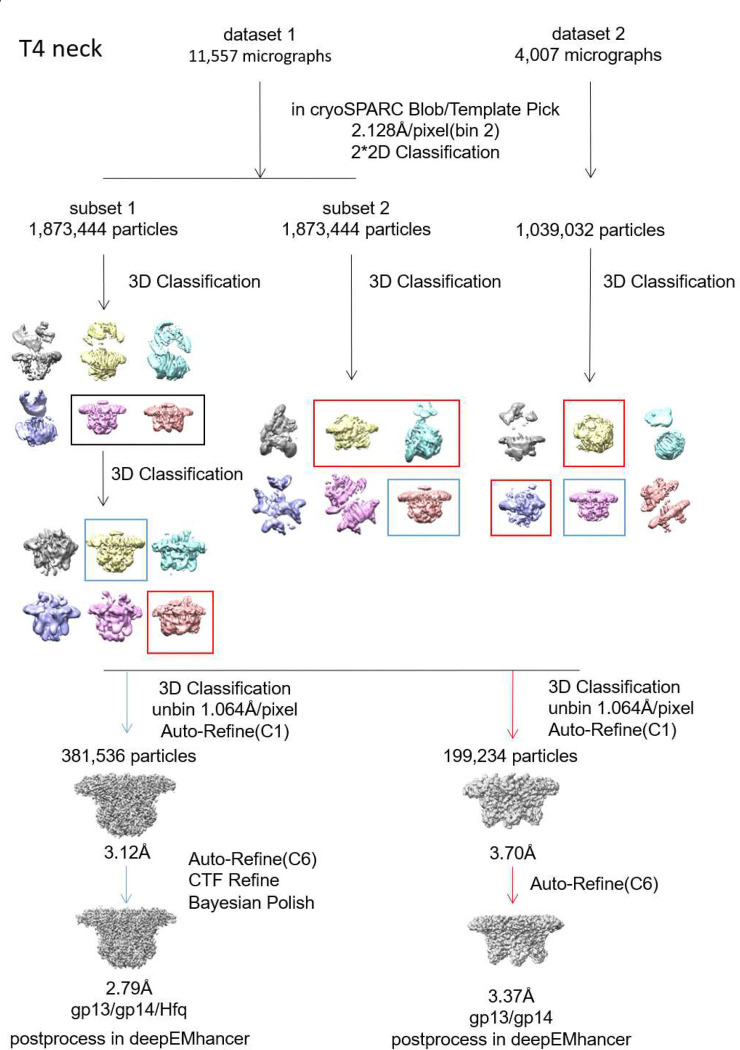
Cryo-EM data processing flowchart of T4 neck (gp13/gp14).

**Extended Data Fig. 3 |:**
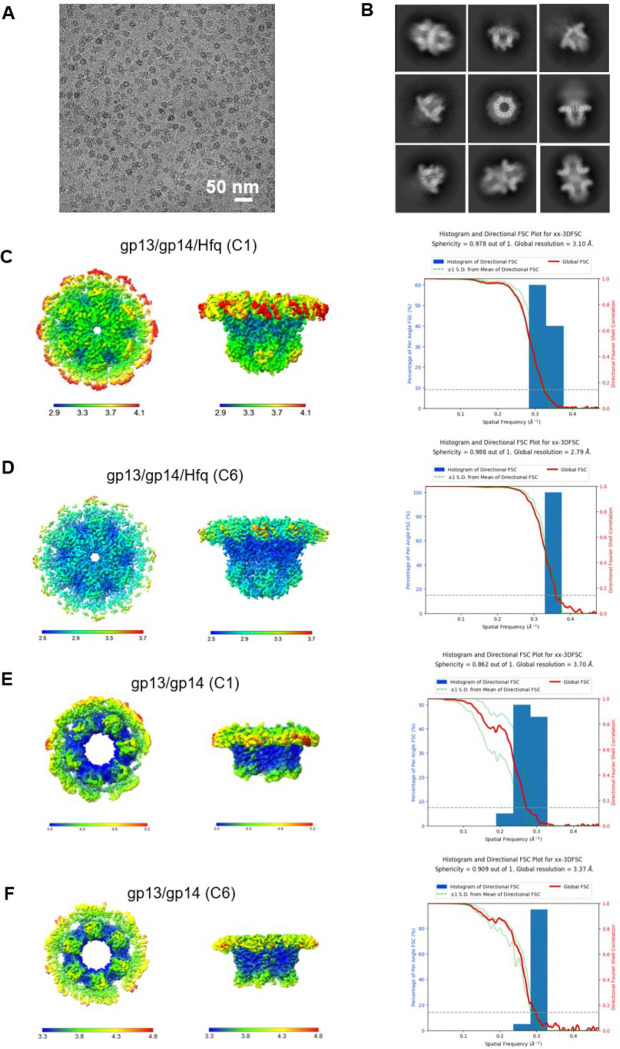
Cryo-EM data processing of the T4 neck (gp13/gp14). (A) Representative cryo-EM micrograph showing gp13/gp14 complexes with a scale bar of 50 nm. (B) Selected 2D class averages, illustrating different views of the particles captured. (C-D) 3D reconstruction of gp13/gp14/Hfq complex without and with C6 symmetry, with the left image showing the structure in surface representation colored by local resolution, and the right plot displaying the Fourier shell correlation FSC curves indicating a global resolution of 3.10 and 2.91 Å, respectively. (E-F) 3D reconstruction of gp13/gp14 complex without and with C6 symmetry, with the left image and right plot showing the local resolution and FSC curve, respectively.

Two types of neck complexes were observed in the cryo-EM images obtained from co-purified gp13 and gp14 fractions; the gp13/gp14 complex comprising ~34% of particles and gp13/gp14/Hfq complex comprising ~66% of particles (Extended Data Fig. 2). Cryo-EM maps were generated for both these complexes without imposing symmetry to 3.7 and 3.12Å resolution, respectively (Extended Data Fig. 3). In the gp13/gp14 complex, gp13 forms a dodecameric ring and interacts with a hexameric gp14 ring creating a central channel. In the gp13/gp14/Hfq complex, the Hfq hexamer attaches to the gp14 hexamer, essentially covering the lower end of the channel (Extended Data Fig. 3C). Notably, the gp13 ring in the gp13/gp14 complex is incomplete, lacking some of the twelve subunits (Extended Data Fig. 3E). In contrast, the gp13 ring in the gp13/gp14/Hfq complex is complete (Extended Data Fig. 3C), suggesting that Hfq stabilizes the structure of the gp13/gp14 complex.

**Fig. 2 |:**
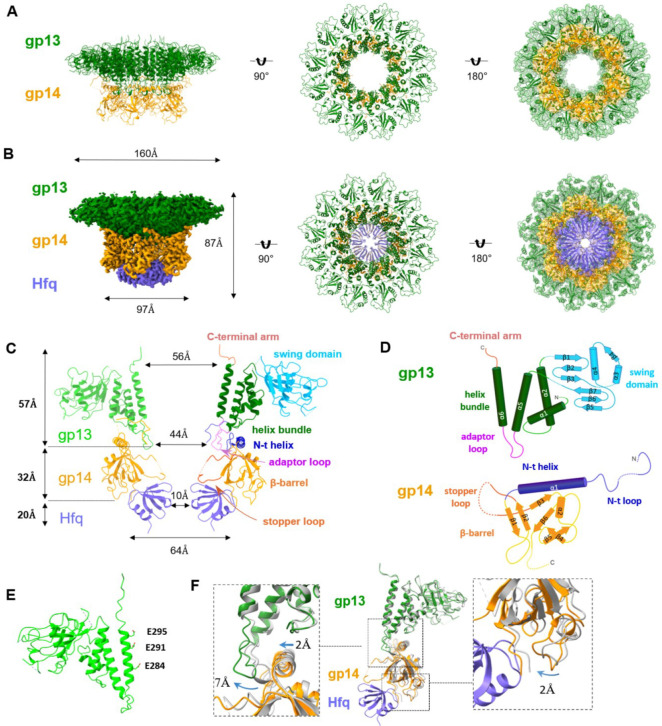
Cryo-EM structures of gp13/gp14 neck complexes. (A) Cryo-EM structures of the gp13/gp14 complex shown in side (left) and top (middle) views, with gp13 in green and gp14 in orange. Right, ribbon representation of gp13/gp14 fitted into the density map. (B) Cryo-EM structures of the gp13/gp14/Hfq complex shown in side (left, cryo-EM map) and top views (middle, ribbon model) with gp13 in green, gp14 in orange, and Hfq in purple. Right, ribbon representation of gp13/gp14/Hfq fitted into the density map. (C) Domain organization in the gp13/gp14/Hfq complex. (D) Schematic representation of gp13 and gp14 secondary structures, with α-helices and β-strands labelled numerically. (E)Three glutamic acids (Glu284, 291, and 295) lining on α6 of gp13, forming a negatively charged surface for DNA passage. (F) Structural comparison of gp13/gp14 (colored in gray) and gp13/gp14/Hfq.

To further improve the resolution of the cryo-EM structures, C6 symmetry was applied and the structures of gp13/gp14 and gp13/gp14/Hfq complexes were refined to 3.47 Å and 2.66 Å resolution, respectively, allowing the building of atomic models (Fig. 2A, B; Extended Data Fig. 2,3; Extended Data Table. 1). The gp13/gp14/Hfq complex has an inverse bowler hat shape, with gp13 serving as the brim, gp14 as the body, and Hfq as the crown (Fig. 2B). The gp13 dodecamer forms a central tunnel with an inner diameter ranging from 56 Å at the top to 44 Å at the bottom (Fig. 2C). The gp13 subunit can be divided into five parts: domain I, a four-helix bundle (residues 3–51, 239–300); domain II, the “swing” domain (residues 52–192); the fibritin-binding domain III (residues 190–239) which is disordered in the current structure but resolved in the virion (Fig. 6; Fokine etal., submitted); the gp14-binding adaptor loop (residues 266–279), and the portal-binding C-terminal arm (residues 301–308) (Fig. 2C and 2D).

**Fig. 3|:**
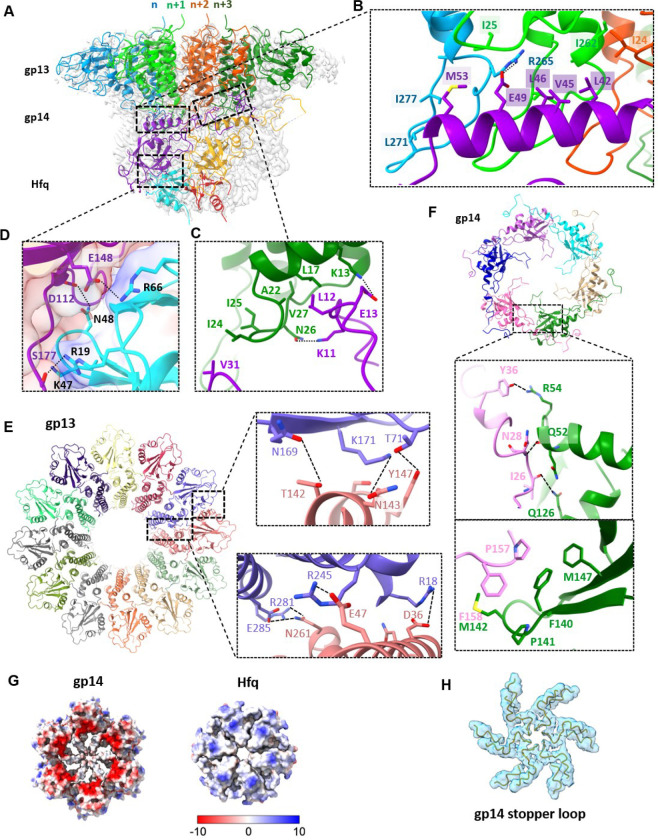
The networks of interactions between gp13, gp14 and Hfq. (A) Ribbon diagram of the gp13/14/Hfq structure fitting into the cryo-EM density map. Four neighboring gp13 subunits are labeled sequentially as n, n+1, n+2, and n+3 and colored in blue, lime, orange red and forest green; Two neighboring gp14 subunits are shown in violet and orange and two neighboring Hfq subunits are shown in cyan and red. (B-C) Close-up view of gp13 and gp14 interfaces. Residues involved in hydrophobic and electrostatic interactions are shown as sticks. (D) Close-up view of the gp14-Hfq interface. Residues involved in salt bridge and hydrogen bonding interactions are shown as sticks. (E) Top view of the gp13 oligomeric ring, with each subunit in a different color. The boxed regions highlight the specific interaction interfaces between monomers. (F) Top view of the gp14 oligomeric ring, with each subunit in a different color. The boxed regions highlight the specific interaction interfaces between monomers. (G) Electrostatic surface representation of gp14 (bottom view) and Hfq (top view), illustrating positively charged (blue) and negatively charged (red) regions. (H) Ribbon diagram of the stopper loop region fitting into the cryo-EM density map of gp20/gp13/gp14/Hfq.

The helix bundle and the swing domains of gp13 form the core and the peripheral ring, respectively. The helix bundle is made of four helices (α1, α2, α5, α6), with α6 forming the central channel. Three glutamic acids (Glu284, Glu291, and Glu295) on α6 face the channel, creating a negatively charged surface for smooth passage of DNA (Fig. 2E). The swing domain, so named because it is observed to swing upwards in the mature T4 virion (Fig), is made of seven β strands and two α helices (β1–7, α3,4) (Fig. 2D). Other than these two main domains, gp13 contains two major loops located before and after α6, named as the adaptor loop and the C-terminal arm, respectively. The 14-aa adaptor loop inserts into gp14, forming extensive interactions (Fig. 3A–3C). The C-terminal arms stretch upward, providing a binding site for gp20. The interaction between adjacent gp13 subunits is mainly mediated by hydrogen bonds between the swing and helix bundle domains (Fig. 3E).

Gp14 consists of an N-terminal loop (residues 5–37), a long helix (residues 38–54), a long stopper loop (residues 89–112), and a core β-strands domain (residues 55–88, 113–177) (Fig. 2C and 2D). The long N-terminal loop and the N-terminal lateral helix (α1) of gp14 wrap around the adaptor loop of gp13, while six core β-strands (β1–6) form an anti-parallel β-barrel. The long loop between β2 and β3 protrudes into the center of the channel. Six such loops, referred to as “stopper loops”, form a gate-like structure that narrows the otherwise open channel to ~10Å (Fig. 3D and H), likely preventing the release of the tightly packed genome from the pressurized capsid. The C-terminal 80 amino acids are invisible, probably due to their flexibility. The loop between β5 and β6 interacts with the adjacent subunits mainly through hydrophobic interactions (I26, N28, Y36 and R54, Q52, Q126). Moreover, residues P157 and F158 form hydrophobic interactions with F140, P141and M147 of the adjacent subunit (Fig. 3F).

Gp13 and gp14 engage in intensive interactions, with one gp14 interacting with four gp13 subunits (n, n+1, n+2, n+3) (Fig. 3A–3C). In addition, the N-terminal loop of gp14 interacts with gp13 (n+3) through both hydrophobic and hydrophilic interactions (Fig. 3C). The N-terminal helix α1 of gp14 spans the adaptor loops of three adjacent gp13 subunits (n, n+1, n+2), forming intensive hydrophobic interactions (M53, L46, V45, L42 from gp14 and I277, L271, I262, I24, I25 from gp13) (Fig. 3B). The interface area between gp13 dodecamer and gp14 hexamer is extensive, 10,675Å^2^, in contrast to the interface area between gp13 subunits (1,725.6Å^2^) or gp14 subunits (927Å^2^). This explains why neither gp13 nor gp14 could form oligomers when expressed alone, but could do so when mixed. A similar phenomenon was also observed in the portal-neck assembly of P22 phage.^40,41^

Notably, the *E. coli* Hfq hexamer fits precisely at the bottom of gp14, forming a stable interaction (Fig. 3G). The Hfq hexamer engages with the gp14 hexamer primarily through electrostatic interactions and hydrogen bonds, involving residues D112, E113, N150, E134, E148, S177 of gp14 and N33, R66, N48, T49, K47, S65, N35, R19 of Hfq, resulting in a substantial buried interface of 3,159.2Å^2^ (Fig. 3D and 3G). Remarkably, the disordered stopper loop residues (106–113) of gp14 become visible only upon Hfq binding, suggesting that Hfq binding stabilizes the stopper loop (Fig. 2E and 3H). Moreover, the insertion of Hfq hexamer constricts the central channel once again, to a diameter of ~10Å, likely functioning as a second genome gate and also reinforcing the first gate to prevent DNA leakage (Fig. 2C).

Furthermore, comparison of the structures of the gp13/gp14/Hfq and gp13/gp14 complexes reveal that Hfq binding leads to further narrowing of the tunnel formed by gp14’s β-barrel and gp13’s adaptor loop by approximately 2Å in radius. Additionally, the adaptor loop of gp13 exhibits a tangential shift of ~7 Å (Fig. 2F).

### Structure of the portal-neck connector complex

**Extended Data Fig. 4 |:**
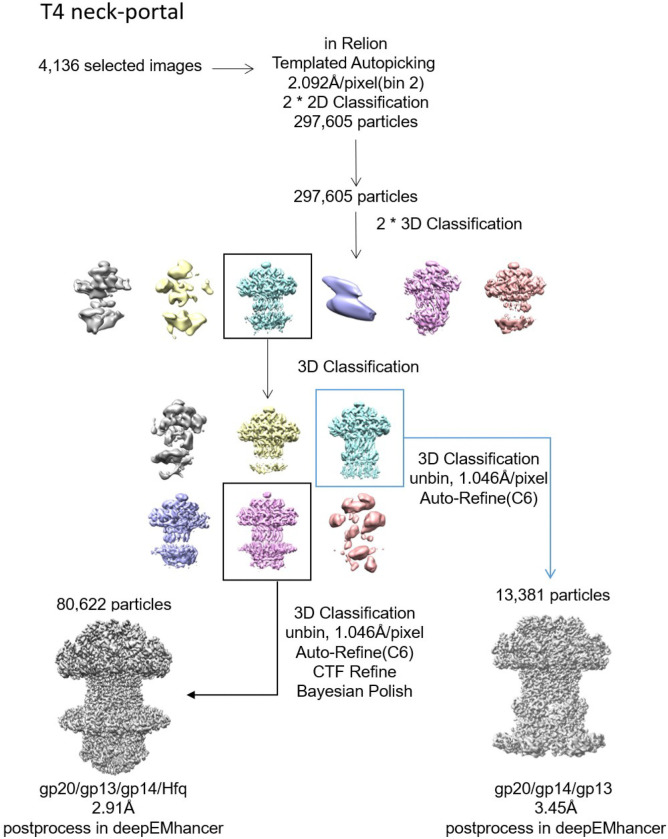
Cryo-EM data processing flowchart of portal-neck complex.

**Extended Data Fig. 5 |:**
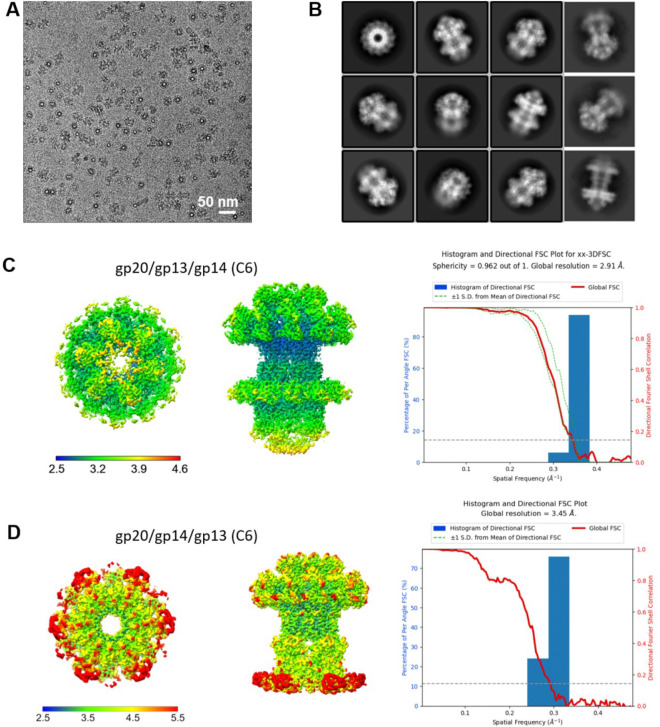
Cryo-EM data processing of T4 portal-neck complex (gp20/gp13/gp14). (A) Representative cryo-EM micrograph showing gp20/gp13/gp14 complexes with a scale bar of 50 nm. (B) Selected 2D class averages of the gp20/gp13/gp14 complexes. (C) 3D reconstruction of gp20/gp13/gp14 (C6) complex, with the left image and right plot showing the local resolution and FSC curve, respectively. (D) 3D reconstruction of gp20/gp14/gp13 (C6) complex.

**Fig. 4 |:**
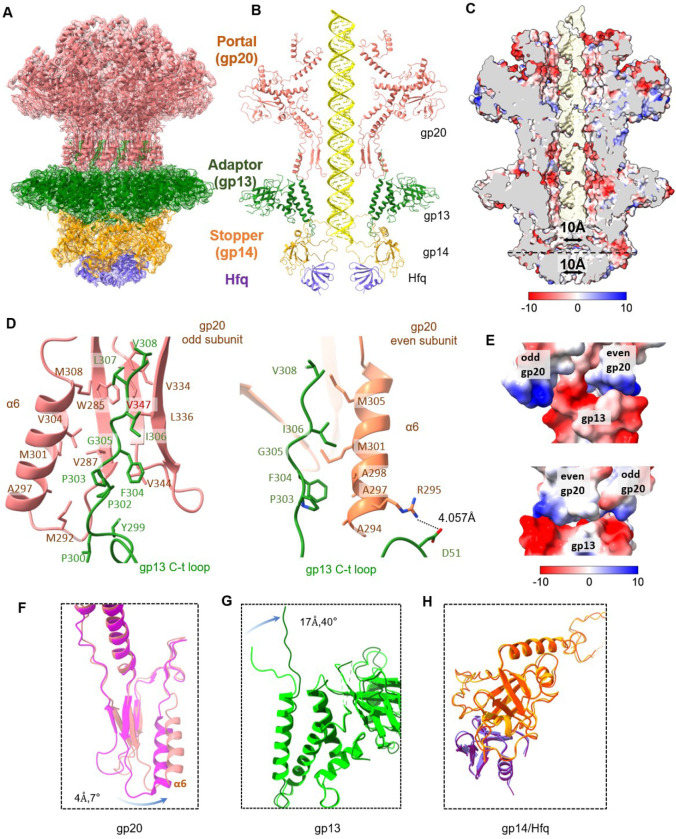
Structural characterization of the gp20/gp13/gp14 complex with Hfq binding. (A) Cryo-EM map of T4 gp20/gp13/gp14/Hfq complex, with gp20 in red, gp13 in green, gp14 in yellow, and Hfq in purple. (B) Ribbon diagram of the gp20/gp13/gp14/Hfq complex. A dsDNA model was modelled into the central tunnel. (C) Electrostatic surface representation of the gp20/gp13/gp14/Hfq complex. Unit on color key: kcal/(mol·e). (D) Interface between the C-terminal arm of gp13 and two neighboring subunits of gp20, highlighted residues involved in hydrophobic interactions and hydrogen bonds. (E) Electrostatic surface representation showing interactions between gp20 subunits with gp13, emphasizing the distribution of charged regions. (F) Structural comparison of apo-gp20 (PDBID: 3JA7, shown in magenta) with gp20 in gp20/gp13/gp14/Hfq complex (shown in pink), showing the rotation of the α6 helix (4.7°) upon complex formation. (G) Structural comparison of gp13 between gp13/gp14/Hfq (light green) and gp20/gp13/gp14/Hfq (dark green) complexes, showing 40° rotation of C-terminal arm of gp13 upon gp20 binding. (D) Structural comparison of gp14/Hfq between gp13/gp14/Hfq and gp20/gp13/gp14/Hfq complexes, indicating no obvious conformational change.

Cryo-EM structural analysis of the *in vitro* assembled gp20/gp13/gp14 complexes revealed that a majority (~90%) of these complexes also contained the Hfq hexamer. Therefore, the structure of the portal-neck connector complex that joins the head and the tail was resolved at 2.91Å with C6 symmetry (Fig. 4A, 4B and Extended Data Fig. 4,5).

**Extended Data Fig. 6 |:**
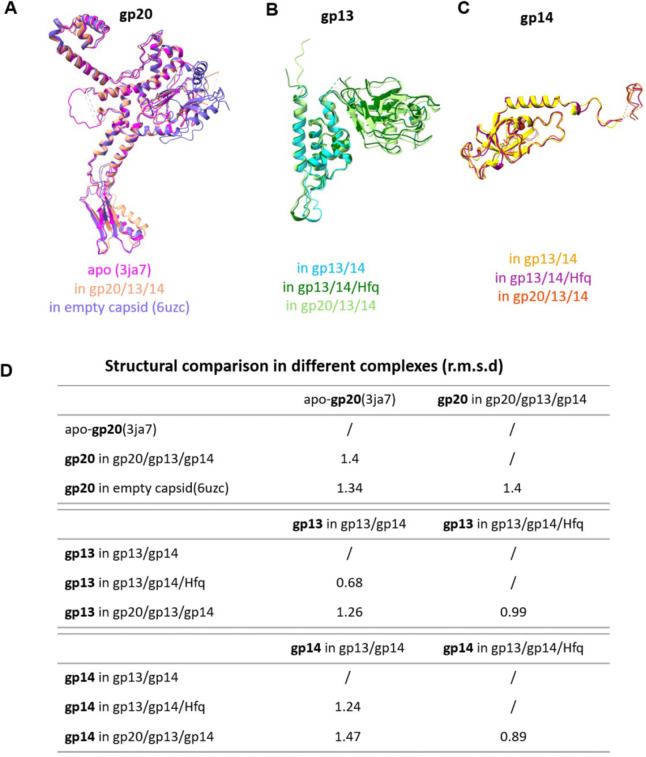
Structural analysis of gp20, gp13, and gp14 conformations in different complexes. (A-C) Superimposition of gp20 (B), gp13 (D) and gp14 (D) structures in different complexes. (D) Table summarizing the structural changes of gp20, gp13, and gp14 in various complexes: apo-gp20 (3ja7), gp20 in gp20/13/14 complex, gp20 in empty capsid (6uzc), and the states of gp13 and gp14 in gp13/gp14, with and without Hfq binding.

The overall structure of the dodecameric gp20 in this complex is similar to that of the recombinant gp20, with a root mean square deviation (r.m.s.d.) of 1.4 Å across 411 (85%) aligned residues (Extended Data Fig. 6A). However, upon gp13 binding, the α6 helix of gp20 clip domain rotates 7° and shifts 4 Å to accommodate the binding of the gp13 (Fig. 4F). The C-terminal arm of gp13 rotates upward by 40° relative to that in gp13-gp14 complex (Fig. 4G), inserting itself between two clip domain α6 helices of adjacent gp20 subunits, and forming extensive hydrophobic interactions with residues on both the helices and with the β-strand of one of the clip domains (Fig. 4D). Furthermore, D51 of gp13 and R295 of adjacent gp20 form salt-bridges (Fig. 4D). Additionally, the negatively charged residues at the C-terminus of gp13’s α2 and α4 helices (E47, D51, E295, D301) engage in electrostatic interactions with positively charged residues of gp20’s α6 helix (Y295, K296, R339) (Fig. 3E). On the other hand, the conformation of the gp14 and Hfq complexes remained unchanged when the neck complex attaches to the portal (Fig. 4H; (Supplementary Movie 1).

### Hfq ensures correct assembly of portal-neck complex

**Fig. 5 |:**
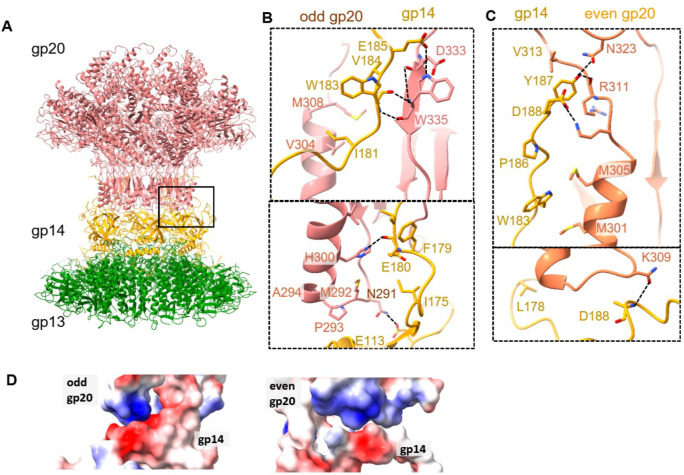
Cryo-EM structure of misassembled gp20/gp14/gp13 complex. (A) Ribbon diagram showing the overall structure of the gp20/gp14/gp13 complex, with gp20 in pink, gp14 in yellow, and gp13 in green. (B-C) Close-up view of the interactions between gp20 (pink) and gp14 (yellow), showing key residues involved in the complex formation. Dashed lines indicate hydrogen bonds and salt bridges. (D) Electrostatic surface representations of the interactions between gp20 with gp14, showing the distribution of positive (blue) and negative (red) charges, showing the electrostatic interactions.

Unexpectedly, we identified a novel class of particles among the portal-neck connector complexes in which gp14, instead of gp13, interacted with gp20 and is positioned between the gp20 portal dodecamer and the gp13 dodecamer (Fig. 5A; Extended Data Fig. 4 and 5). Notably, these gp20/gp14/gp13 complexes are devoid of Hfq and constituted ~14% of the portal-neck complexes (Extended Data Fig. 4). In this structure determined at 3.45Å resolution, the C-terminal arm of gp14, which normally makes extensive interactions with gp15 subunits, inserts between the clip domains of adjacent gp20 subunits. Similar to the gp20/gp13 complex, gp20 and gp14 mainly interact through hydrophobic interactions and hydrogen bonds (K309, N291, N323, R311 of gp20 and D188, A110, Y187 of gp14) (Fig. 5B and 5C). Furthermore, residue K309 of gp20 forms a salt-bridge with D188 of gp14. Additionally, electrostatic interactions are also observed between the positively charged clip domain of gp20 (residues of R295, K296, H300 and H303) and the negatively charged C-terminus of gp14 (Fig. 5D).

To further investigate this unexpected phenomenon, and if Hfq played a role in assembling the correct portal-neck complex (gp20/gp13/gp14), we produced recombinant Hfq protein and mixed it in excess first with gp14. We then added gp13 followed by the portal protein. Cryo-EM analysis of the assembled complexes revealed that only the correctly assembled gp20/gp13/gp14 complexes were formed, and no mismatched gp20/gp14/gp13 complexes were observed in the presence of Hfq (Extended Data Fig. 6). These results suggest that Hfq, through binding to gp14, may inhibit gp14’s interaction with gp20, thereby facilitating the correct assembly of the portal-neck connector complexes. Additionally, since Hfq pre-exists as a hexamer during phage infection, this interaction might also help initiate neck assembly.

### Conformational changes in portal-neck connector complexes

**Fig. 6 |:**
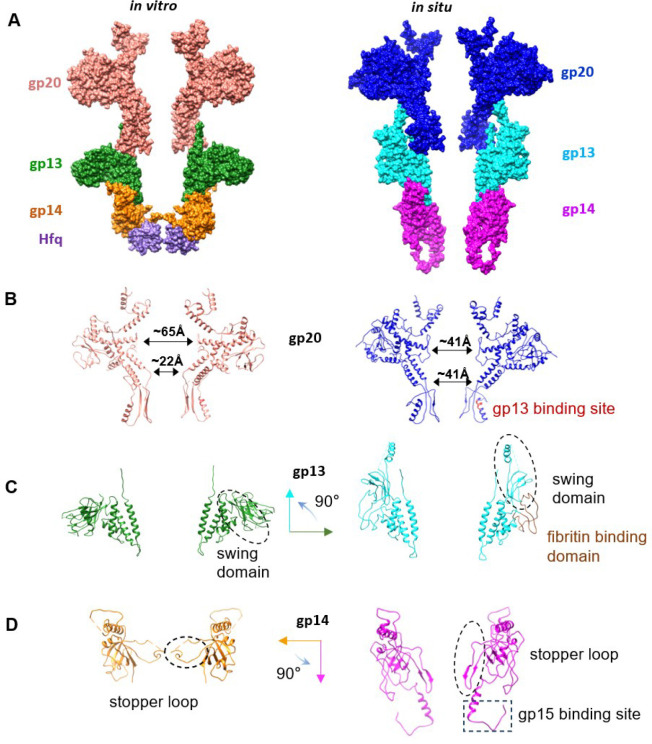
Structural comparison of *in vitro* and *in situ* portal-neck complexes. (A) Left: *in-vitro* structure model of gp20/gp13/gp14/Hfq. Right: *in-situ* structure model of gp20/gp13/gp14 complex. (B-D) Structure comparison of in-vitro (left) and in-situ (right) gp20., gp13, gp14 proteins.

Comparative structural analysis showed that the structures of gp20, gp13 and gp14 in the different *in vitro* assembled complexes are similar with r.m.s.d. ranging from 0.68–1.47 (Extended Data Fig. 6). However, comparison of the *in vitro* assembled gp20/gp13/gp14/Hfq complex with the *in situ* gp20/gp13/gp14 atomic model of phage virion (Fig. 6; Fokie et al., submitted) shows dramatic conformational changes in each of the neck components, which lead to a dynamic genome retention mechanism (Fig. 6A). Upon completion of headful packaging, a global conformational change occurs in the portal structure and the portal is pushed down by the pressure of the packaged genome exposing the “stem” and “wing” regions of portal for gp13 binding (Fig. 6B). After initial binding with the clip domain, as seen in the current structure, a major conformational transition occurs in the gp13 dodecamer. The swing domain flips upward by approximately 90° and its “anchor” region (aa 90–157) interacts with the now exposed stem and wing domains of the portal and the periphery domains of the major capsid protein gp23 (Fig. 6C). Furthermore, residues 193–238 which are not resolved in the current structure, model into domain III which binds to “fibritin”, a trimer of gpWac (*w*hisker *a*ntigen *c*ontrol) (Fig. 6C). Twelve domains III of the gp13 dodecamer interacting with fibritins assemble twelve fibers around the neck, six as “whiskers” and the other six as “collar”. Later in virion assembly, these neck fibers interact with the long tail fibers (LTFs) in ‘up’ conformation in the pre-infection stage.

At the other end of the portal-neck connector, gp14 also undergoes a large conformational change. The C-terminal regions of gp14 (aa 178–256), which are disordered in the current structure, form new and extensive interactions with gp15 subunits (residues 203–245), while the β-barrel stopper loops rotate downward by ~90 ° to dock with the tail sheath terminator protein gp15 located at the tip of the tail (Fig. 6D). These conformational changes open the gp14 genome-gate, while also ejecting the Hfq hexamer (Supplementary Movie 2).

These analyses reveal that the current structures represent intermediates trapped by *in vitro* assembly prior to tail attachment, thus preserving the structure of a transient genome plug formed to leakproof the head and retain the packaged genome while handing it over to the tail.

## Discussion

One of the least understood mechanisms in large icosahedral virus assembly is how the pressurized viral genome inside a capsid is contained during the post-packaging transactions^42^. After encapsidating a headful genome, packaging has to be terminated and the motor must be expelled, in order for the neck proteins to assemble followed by tail attachment, all occurring in rapid succession. With ~25–35 atm pressure inside the packaged head, expulsion of even a few base pairs of DNA during these transactions could result in abortive assembly resulting in a non-infectious virus particle. Despite many phage and viral structures solved by cryo-EM, the dynamics of assembly and the conformational transitions that allow full retention of the genome at this critical juncture remain unknown. Here, our structural and biochemical studies using the *in vitro* assembled portal-neck connector intermediates reveal a novel double genome-gate structure involving a host component, which creates a near perfect seal of the encapsidated viral genome.

Though a genome-gate has been implicated in other phages, such as SPP1^31,43^, the double gate structure observed in T4 by co-opting the host protein Hfq is distinctive. No such structure has been reported in any phage or viral system. Hfq, being an abundant, hexameric, nucleic acid-binding protein, is an ideal choice for this function^34,35^. Indeed, the negatively charged regions at the bottom surface of gp14 hexamer perfectly lock-in with the positively charged surface of the Hfq hexamer to create such a structure (Fig. 2). With this second genome-gate reinforcing the first gate formed by the gp14 hexamer, a tight plug is established, essentially sealing off the pressurized capsid (Fig. 2C).

**Extended Data Fig. 7 |:**
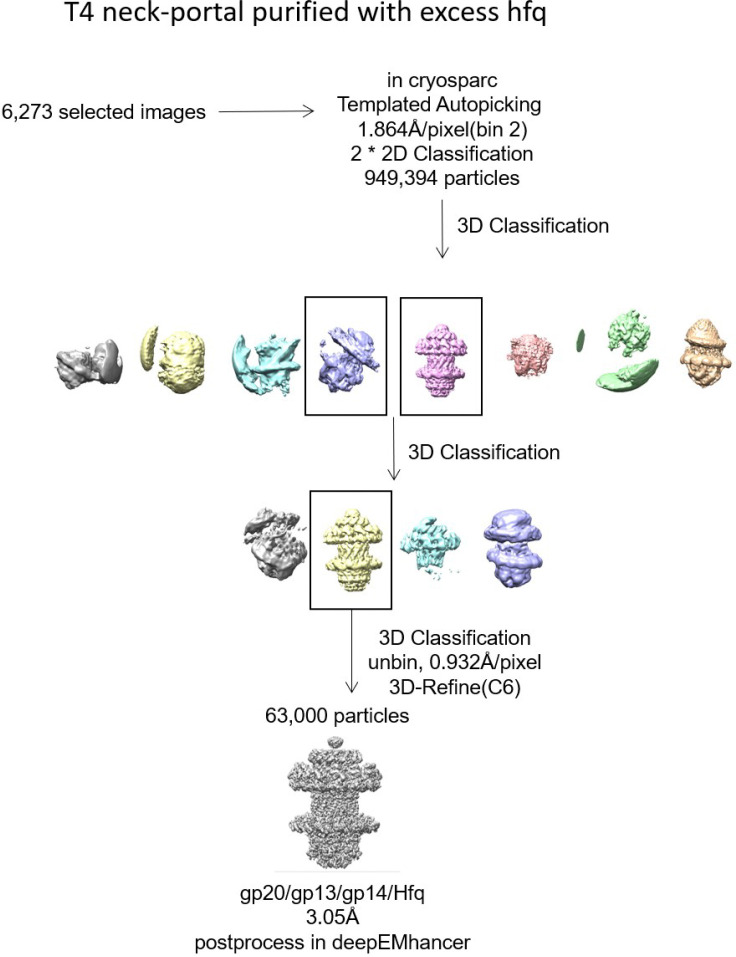
Cryo-EM data processing flowchart of portal-neck complex purified with excess Hfq. No misassembled complex (gp20/gp14/gp13) was observed.

Hfq also serves a second and complementary role, which is also novel and important. It maximizes the fidelity of the neck assembly by channeling gp14 to the correct assembly pathway, by preventing nonspecific interactions with the gp20 portal dodecamer, which would otherwise lead to abortive assembly. While ~14% such abortive structures were formed in the *in vitro* assembly reaction (Extended Data Fig. 7), addition of excess Hfq eliminated these mis-assemblies (Fig. 4A). The network of electrostatic interactions between the gp14 and Hfq hexamer surfaces once again ensures that gp14 remains in the correct assembly state, i.e., interact with the tail (gp15), not with the portal.

**Fig. 7 |:**
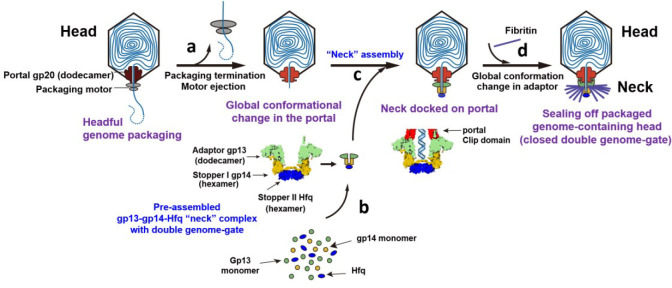
Mechanism of genome retention in bacteriophage T4. After headful genome packaging, a global conformational change (a) in the dodecameric portal structure ejects the packaging motor and exposes binding sites for neck assembly. A pre-assembled gp13 dodecamer (adapter)-gp14 hexamer (stopper I)-Hfq hexamer (stopper II) neck complex (b) docks on the portal clip domains through gp13’s C-terminal arms (c). This induces a global conformational change in gp13 locking-in the neck-portal complex, sealing off the packaged genome-containing head with a double genome-gate, and attaching fibritin fibers (d).

These and other structural analyses lead to a conformation-driven genome retention mechanism in phage T4 (Fig. 7). Our *in vitro* assembly data suggest that neither gp13 nor gp14 alone can oligomerize but together they do, forming a gp13 dodecamer/gp14 hexamer complex. However, this complex is unstable losing some of the gp13 subunits in the dodecamer when Hfq is not present (Extended Data Fig. 3E). But with Hfq bound to gp14, a complete and stable gp13 dodecamer-gp14 hexamer-Hfq hexamer complex is efficiently assembled (Fig. 7b) and the gp13’s unstructured C-terminal segments are stretched upwards and exposed on the surface, being ready to dock onto the portal (Fig. 2A–C).

Since the gp13 binding sites are not yet available during packaging, and since the packaging motor is still attached to the portal^13^, premature docking is prevented and the neck complex awaits packaging to complete. Once the head is full and packaging terminated, the portal switches conformation ejecting the packaging motor and it is pushed down exposing the gp13 binding sites (Fig. 7a). The gp13-gp14-Hfq neck complex then docks onto the portal, first through insertion of the gp13 C-terminal segments into the portal clip domains, as observed in the current portal-neck-connector intermediate formed *in vitro* when the portal clip domains are fully exposed (Fig. 5D). This then triggers a conformational change causing the gp13’s swing domain to flip upwards by 90° and embrace the stem and wing domains of the portal (symmetry-matching interactions) while another segment interacts with the “P” (periphery) domains of the major capsid protein gp23 (symmetry-mismatched interactions), as observed in the *in situ* portal-neck connector structure (Fig. 6C). Additionally, other previously unstructured regions of gp13 remodel into binding domains that capture the gpWac fibritin trimers. These interactions lead to the twelve fibers decorating the neck, six of them forming the propeller-shaped collar and the other six facing down forming the whiskers^44^. Thus, a stable and intricately woven head-portal-neck structure is formed with the neck’s channel sealed off by the double genome-gate at the bottom. Inside this structure resides the packaged genome with the last-packaged DNA stopped by the genome gate (Fig. 7c, d). The viral genome thus is safely sequestered inside this structure, which awaits the next step in virion assembly, tail attachment. Additional conformational changes would then follow when tail attaches leading to genome positioning in the innermost tunnel of the virion.

To our knowledge, this is the first detailed mechanism for viral genome retention in icosahedral phages and viruses, which emerged through examination of the structures of the *in vitro* assembled intermediates in comparison with the finished virion. Particularly eye-opening is the dynamic conformation-driven transitions in every component of the portal-neck connector complex as the virus undergoes post-packaging assembly and morphogenesis, which previously was assumed to be simple binding steps. Clearly, the structures of the finished phages alone^20,29,45,47–51^ could not reveal such conformational transitions^20,29,44–49^. Given the structural and functional parallels among large icosahedral viruses, the basic mechanism is likely preserved in other phages and viruses, which might also present a new target for antiviral discovery. Additionally, our studies reveal a unique feature T4 had evolved, transiently hijacking host Hfq to streamline the assembly process, as well as to ensure high retention efficiency. Though not essential, such an engagement of an accessory host component greatly enhances T4 phage production, and thus its fitness to survive in an extremely competitive phage universe.

## Methods

### Protein expression and purification

The full-length gp13 gene (NC_000866.4), gp14 (NC_000866.4) and Hfq(NC_000913.3) were synthesized (GeneScript) and inserted into the pET-29a, pET28b and pET-28a vectors with N-terminal His tag, N-terminal Strep II tag(Hfq) respectively. The protein was expressed in *E. coli* BL21(DE3) grown at 37°C, induced by 0.5mM isopropyl β-D-1-thiogalactopyranoside (IPTG) at an optical density (OD) of 0.6~0.8 at 16°C. After 4 hours, the cells were harvested by centrifugation at 5,000 rpm and resuspended in lysis buffer (20mM Tris, pH8.0, 200mM NaCl, 20mM imidazole, 1/500 Supernuclease S (Tiandirenhe), 1/10000 Protease Inhibitor Cocktail (Roche). The mixture was then centrifuged at 40,000 × g for 20 min at 4 °C and removed fragments by 0.22μ filter (Millipore USA). The sample was loaded into Ni Smart Beads 6FF (Tiandirenhe) equilibrated in buffer A (20mM Tris, pH8.0, 500mM NaCl), and eluted with buffer A containing 500mM imidazole. The protein was then purified by Superose 6 column (GE Healthcare) with buffer B (20mM Tris, pH8.0, 500mM NaCl, and 1mM TCEP). The elution volume indicated that gp13 and gp14 were monomeric in solution. For further studies, we mixed gp13 and gp14 lysates together from the very beginning in bacterial sonication and centrifugation, and then we used the same purification protocol described above. N-His tagged gp20 (residues 74–524)(NC_000866.4) and untagged gp22 were cloned into the pET-Duel vector for co-expression and purified as previously described [1]. Finally, we mixed gp13/gp14 neck complex and portal protein gp20 together and incubated for 30 min, and then the mixture was concentrated with 50,000 Da Ultra tube (Millipore) into 0.5 ml for size exclusion chromatography (Superose 6^™^ Increase 10/300 GL, Cytiva).

### Identification of proteins in the neck complexes by LC/CL-MS analysis.

The gp13, gp14, and Hfq bands were excised from the gel and digested with Trypsin at 37℃ for 4 h. The concentrated peptides were analyzed by Orbitrap Fusion^™^ Lumos^™^ Tribrid^™^ Mass Spectrometer (Thermo Fisher Scientific). Original data was analyzed by Thermo Proteome Discoverer 2.5.0.400 followed by searching for specific protein sequences in data bank.

### Phage yield in Hfq-minus *E. coli*

*E. coli* strains NM22565 (Hfq-plus) and NM22540 Dhfq::cat-sacB (Hfq-minus) were grown to mid-logarithmic phase (~2 × 10^8^ CFU/mL) in Luria-Bertani (LB) medium at 37°C with shaking at 250 rpm. The cells were infected with wild-type (WT) T4 phage at a multiplicity of infection (MOI) of 0.1 and incubated at 37°C for 5 minutes. Immediately afterward, serial 10^2^-, 10^4^-, and 10^6^-fold dilutions were prepared in LB medium. The remaining infection mixture (non-diluted) was treated with chloroform to lyse the cells and quantify unbound phage. At 10, 20, 30, 40, and 60 minutes post-infection, aliquots were taken from the 10^6^-fold dilution and subjected to plaque assays by the soft agar overlay method using *E. coli* B40. Burst size was calculated as plaque-forming units (PFU) produced per infected cell.

### Construction of Hfq^+^ T4 Phage

The Hfq^+^ T4 phage was generated using phage CRISPR-mediated gene insertion, as described previously^50^. Briefly, *E. coli* B40 cells were co-transformed with a donor plasmid containing *E. coli hfq* gene flanked by T4 genomic sequences in the *SegF* region and a CRISPR-Cas12a-SegF spacer plasmid. The cells were infected with T4-Soc-del phage. Recombinant phages were selected by counter-selection in *E. coli* B40 expressing the Cas12a-SegF spacer. PCR analysis confirmed successful insertion of the *hfq* gene under the control of Soc promoter. To verify Hfq expression by the constructed *hfq^+^* T4 phage, *E. coli* NM22565 (*hfq*^−^) cells were grown to mid-log phase in LB medium at 37°C (~10^8^ CFU/mL) and infected with the Hfq^+^ phage at an MOI of 5. After 5 minutes of incubation at 37°C, the culture was super-infected with an additional dose of *hfq*^+^ phage (MOI 5) to prevent lysis. At 30 and 60 minutes post-infection, ~10^8^ CFU of bacteria were collected for SDS-PAGE and Western blotting using Hfq polyclonal antibodies (see below). The data show that Hfq is well-expressed from *hfq*^+^ phage during phage infection.

### Hfq Western blotting

*E. coli* strains [B40, NM22565 (Hfq-plus) and NM22540 Dhfq::cat-sacB (Hfq-minus)] were grown to mid-logarithmic phase in LB medium at 37°C with shaking at 250 rpm. Bacterial cells (approximately 10^8^ CFU) were harvested by centrifugation at 5,000 × g for 5 minutes, resuspended in 200 μL 1X SDS-PAGE loading buffer (50 mM Tris-HCl pH 6.8, 2% SDS, 10% glycerol, 1% β-mercaptoethanol, 0.02% bromophenol blue), and denatured by boiling for 10 min. The samples were then electrophoresed on a 4–20% gradient Mini-PROTEAN TGX precast gel (Bio-Rad) and transferred to nitrocellulose membranes using a Trans-Blot Turbo transfer system (Bio-Rad). Membranes were blocked with 5% BSA in PBS-T (PBS containing 0.05% Tween-20, pH 7.4) for 1 hour at room temperature with gentle shaking, then incubated overnight at 4°C with anti-HFQ rabbit polyclonal antibody at 1:1,000 dilution in 5% BSA/PBS-T. After five 5-minute washes with PBS-T, membranes were incubated with HRP-conjugated goat anti-rabbit secondary antibody (1:10,000, Abcam) for 1 hour at room temperature. Following five additional PBS-T washes, stained protein bands were visualized using an enhanced chemiluminescence substrate (BioRad) and imaged using a Bio-Rad Gel Doc XR+ imaging system with exposure times optimized for each blot.

### Cryo-EM sample preparation and data collection.

All samples were evaluated by negative stain before preparing cryo-grids. 5μL purified samples were applied to the glow-discharged copper grids (Electron Microscopy China) for 1 min then blotted with filter paper. The grids were then negatively stained with 2% (w/v) uranyl acetate, blotted and air-dried. Images were recorded with Talos L120C TEM (Thermo Fisher Scientific) equipped with a CCD camera at a nominal magnification of ×73000 or ×92000, defocus between −0.5 μm and −3.5 μm.

All cryo-grids were prepared with the same condition. Here, we took gp20/13/14 as an example and more details are shown in Table 1 of Supplemental Information. Quantifoil holey carbon grids (R20/20 Ni-Ti-Au300) were glow-discharged under the atmosphere of argon and oxygen mixture. 3μL sample containing 0.05 % β-OG was loaded on the grids, and then blotted and vitrified using a Vitrobot Mark IV (Thermo Fisher Scientific). All cryo-EM data were collected at the Center for Cryo-Electron Microscopy (Fudan University) with Titan Krios TEM (Thermo Fisher Scientific) operated at 300kV, equipped with BioQuantum energy filter (Gatan).

### Cryo-EM images processing

For gp13/14/Hfq complex, a total data set of 12,169 images were collected with the defocus range of −1.2 μm to −2.2 μm, in super-resolution mode (magnification 81,000X) using K3 Summit direct electron detection device with a physical pixel size of 1.064Å/pixel. The total exposure time was 3s with 40 frames giving an accumulated dose of 58.3 e^−^/Å^2^. Automated data acquisition was performed with SerialEM software through the beam-image-shift method ^51^. More details are listed in Table 2 of Supplemental Information. All super resolution images were binned 2, dose weighted, and motion corrected using MotionCor2 ^52^ and subsequent CTF (contrast transfer function) of micrographs was estimated using Gctf ^53^. Bad images were excluded upon ice condition, defocus range and estimated resolution. Remaining 11,557 good images were imported into cryoSPARC ^54^ for further patched CTF-estimating, blob-picking and 2D classification. Good 2D classes were selected as the template for template picking. From the 2D classes, good particles from blob-picking and template-picking were merged and deduplicated. The whole particle stacks were separated into subsets to accelerate processing. 3D classification in Relion of the first subset of 1,873,444 particles shows three different conformations: gp13/gp14-gp14/gp13, gp13/gp14-Hfq and gp13/gp14, the latter two conformations were selected and merged and after another round of 3D classification, followed by 3D auto-refinement with C6 symmetry, CTF Refinement and Bayesian Polish, a map of 2.66 Å gp13/gp14/Hfq was obtained from 607,345 particles. The whole dataset was used for iterative 3D classification to yield a 3.47Å map of gp13/gp14complex.

The gp20/gp13/gp14/Hfq dataset was collected at magnification 130,000x using K2 Summit direct electron detection device with a physical pixel size of 1.046Å/pixel, total exposure time 8s, 36 frames, at an accumulated dose of 53 e^−^/Å. A total of 4,252 images were collected and 4136 good ones were selected. Particles were Laplacian and templated autopicked in Relion, and 297,605 particles were used for iterative 3D classification. The best resolved classes were selected and yielded a 2.96Å map of gp20/gp13/gp14/Hfq complex.

The reported resolutions are all based on the gold-standard Fourier shell correlation (FSC) 0.143 criterion. All the visualization and evaluation of 3D density maps were performed with UCSF Chimera ^55^. The above procedures of data processing are summarized in (Extended Data Fig. 2, 4 and 7). These sharpened maps were generated by DeepEMhancer^56^.

### Model building and refinement

Model building of gp13/14 was performed de-novo in COOT^57^, while Hfq was fitted and refined using previous crystal model (PDB:1HK9). After structure refinement in PHENIX^58^, the model was fit into the gp20/gp13/gp14/Hfq map. Gp20 was built from the crystal model (PDB:3JA7) and manually adjusted in COOT. Statistics associated with data collection, 3D reconstruction and model refinement can be found in Extended Data Table 1.

### Interface calculation

The interface is calculated using PISA: the interface area equals the difference in total accessible surface areas of isolated and interfacing structures divided by two.

## Extended Data

**Extended data Table 1.** Cryo-EM data collection and structure refinement statistics.

	gp13/gp14	gp13/gp14/Hfq	gp20/gp13/gp14/Hfq	gp20/gp14/gp13

**Data collection and processing**				

Magnification	130,000		130,000	
Voltage (kV)	300		200	
Total dose (e^−^/Å^2^)	58.3		53	
Defocus range (μm)	−1.2~−2.2		−1.2~−2.2	
Pixel size (Å)	1.064		1.046	
Symmetry imposed	C6		C6	

Final particles (no.)	199,234	381,536	80,622	13,381

Map resolution (Å)	3.37	2.79	2.91	3.45

**Refinement**				

R.m.s. deviations				

Bond lengths (Å)	0.001	0.001	0.002	0.001

Bond angles (°)	0.407	0.397	0.053	0.384

**Validation**				

Clashscore	6.91	5.98	7.31	7.66

Rotamer outlier (%)	3.87	2.59	3.46	4.86

Ramachandran plot				
Favored (%)	91.97	94.81	95.69	94.73
Allowed (%)	8.03	5.19	4.31	5.27
Disallowed (%)	0.00	0.00	0.00	0.00

**EMDB**	63387	63388	63389	63390

**PDB**	9LU4	9LU5	9LU6	9LU7

## Supplementary Material

Supplementary Files

This is a list of supplementary les associated with this preprint. Click to download.

• movie1.mp4

• movie2.mpg

## Data Availability

Coordinates and electron density maps associated with data reported in this manuscript were deposited to the Electron Microscopy Data Bank (EMDB) and Protein Data Bank (PDB) with accession numbers EMD-63387 and PDB 9LU4 (gp13/gp14), EMD-63388 and PDB 9LU5 (gp13/gp14/hfq), EMD-63389 and PDB 9LU6 (gp20/13/14/hfq) and EMD-63390 and PDB 9LU7 (gp20/gp14/gp13).
